# A new species in the genus *Acartia* Dana, 1846 (Crustacea, Copepoda, Calanoida, Acartiidae) from the South Pacific coastal waters of Nadi Bay, Fiji

**DOI:** 10.3897/zookeys.893.38369

**Published:** 2019-12-02

**Authors:** Seunghan Lee, Ho Young Soh, Wonchoel Lee

**Affiliations:** 1 Biodiversity Institute, Marine Act Co., Seoul 04790, Korea Biodiversity Institute Seoul South Korea; 2 Department of Environmental Oceanography, Chonnam University, Yeosu 59616, Korea Chonnam University Yeosu South Korea; 3 Department of Life Science, Hanyang University, Seoul 04763, Korea Hanyang University Seoul South Korea

**Keywords:** *
Odontacartia
*, planktonic copepod, mtCOI, taxonomy, South Pacific

## Abstract

A new species in the genus *Acartia*, *Acartia nadiensis***sp. nov.**, is described from Fijian coastal waters. This species belongs to the subgenus Odontacartia based on the following morphological features: presence of a rostral filaments, a pointed process on the last prosomite, a serrated terminal spine on female P5, and the absence of a protrusion on the basis of the male right P5. This new species can be differentiated from its congeners by the combination of the absence of a spine on the first segment of the antennules, the short outer seta of female P5, and a medial spine on the exp-2 of the left male P5. Phylogenetic analyses using mitochondrial COI partial sequences show that the new species is distinct from its congeners.

## Introduction

The genus *Acartia* Dana, 1846 is one of the most dominant groups of planktonic copepods and has a worldwide distribution in estuarine, coastal, and even oceanic waters (Bradford 1976; Walter and Boxshall 2019). Sixty-five species in this genus have been reported from various locations ranging from tropical to polar regions (Soh et al. 2013; Razouls et al. 2019; Srinui et al. 2019), and these species have been allocated to six subgenera: *Acartiura* Steuer, 1915, *Euacartia* Steuer, 1915, *Hypoacartia* Steuer, 1915, *Acanthacartia* Steuer, 1915, *Odontacartia* Steuer, 1915, and *Acartia* (= *Plankacartia*) Dana, 1846 (Steuer 1915, 1923). Among these subgenera, the subgenus Odontacartia contains 13 species: *Acartia amboinensis* Carl, 1907; *A. australis* Farran, 1936; *A. bispinosa* Carl, 1907; *A. bowmani* Abraham, 1976; *A. centura* Giesbrecht, 1889; *A. edentata* Srinui, Ohtsuka & Metillo, 2019; *A. erythraea* Giesbrecht, 1889; *A. japonicus* Mori, 1940; *A. lilljeborgi* Giesbrecht, 1889; *A. mertoni* Steuer, 1917; *A. ohtsukai* Ueda & Bucklin, 2006; *A. pacifica* Steuer, 1915; and *A. spinicauda* Giesbrecht, 1889.

During a survey of the diversity of planktonic copepods in Fijian waters, we collected an undescribed species of the genus Acartia that clearly belongs to the subgenus Odontacartia. In this study, we describe the morphological characters of the new species from Nadi Bay, Fiji. Partial mtCOI sequences were also obtained and compared with related species to determine if this new species is also genetically distinct from its congeners.

## Materials and methods

### Sample collection and identification

Specimens were collected from Nadi Bay, Fiji, using a 100 μm mesh plankton net having a 30 cm diameter mouth, and then preserved in 99% ethanol. Specimens were dissected in lactic acid, and mounted on slides with lactophenol. Preparations were sealed with transparent nail varnish. All drawings were prepared using a drawing tube attached to an Olympus BX51 differential interference contrast microscope. For scanning electron microscope (SEM) preparation, specimens were dehydrated in a series of graded ethanol solutions, then placed in isoamyl acetate, critical point dried, mounted on stubs, coated in platinum, and observed under a Hitachi S4700 field-emission electron microscope at Eulji University, Seoul, Korea. Descriptive terminology was adopted from Huys and Boxshall (1991).

### DNA extraction and amplification

For DNA extraction, ethanol was removed from fixed specimens (99% EtOH) by washing with distilled water, and DNA was extracted using a tissue DNA purification kit (COSMO GENETECH, Co. Ltd, Korea). DNA was extracted from individual specimens. mtCOI DNA was amplified in 20 μl reaction volumes containing extracted tissue DNA and primers LCO-1490 (5'-GGT CAA CAA ATC ATA AAG ATA AAG ATA TTG G-3') and HCO-2198 (5'-TAA ACT TCA GGG TGA CCA AAA AAT CA-3') (Folmer et al. 1994). PCR conditions comprised initial denaturation at 94 °C for 5 min, followed by 40 cycles of denaturation at 94 °C for 1 min, annealing at 46 °C for 2 min, and extension at 72 °C for 3 min. This was followed by a final extension step at 72 °C for 10 min. PCR products were evaluated by electrophoresing amplification products on 1% agarose gel containing ethidium bromide. Purification of amplified products was performed using a PCR purification kit (COSMO GENETECH Co. Ltd, Korea), and both strands were sequenced using an ABI 3730XL sequencer (COSMO GENETECH Co. Ltd, Korea).

### Phylogenetic analysis

Sequences were aligned and edited using CLUSTAL W (Thompson et al. 1994) within MEGA6 (Tamura et al. 2013). For the phylogenetic analysis, three *Acartia* species (*A. erythraea*, *A. japonica*, and *A. ohtsukai*) belonging to the subgenus Odontacartia were collected from South Korea and Japan for this study (Table 1). Sequences of *A. pacifica* and *A. spinicauda* were obtained from the NCBI database for comparison. Phylogenetic analysis and pairwise distance analysis were conducted using MEGA6 software using neighbor-joining and minimum-evolution algorithms, respectively, and the Tamura-Nei model of sequence evolution was applied (Tamura and Nei 1993). Codon positions were set as follows: 1^st^ + 2^nd^ + Noncoding. All positions containing gaps and missing data were eliminated. Acartia (Acartiura) omorii Bradford, 1976 was used as outgroup.

**Table 1.** List of species analyzed for molecular comparison.

Species	Locality	GenBank no.	References
A. (Odontacartia) erythraea	Mokpo, Korea	MN603769–MN603773	Present study
A. (Odontacartia) japonica	Okinawa, Japan	MN603774	Present study
A. (Odontacartia) nadiensis	Nadi Bay, Fiji	MN603766–MN603768	Present study
A. (Odontacartia) ohtsukai	Busan, Korea	MN603775–MN603777	Present study
A. (Odontacartia) pacifica	Nakajima Island, Japan	KC287267	Bucklin and Blanco-Bercial 2014
	Nakajima Island, Japan	DQ071177	Ueda and Bucklin 2006
A. (Odontacartia) spinicauda	Xiamen waters, China	DQ665253–DQ665254	Liu et al. 2006
A. (Acartiura) omorii	Gwangyang Bay, Korea	MN603778	Present study

## Systematics

### Order Calanoida G. O. Sars, 1903

#### Family Acartiidae G. O. Sars, 1900

**Genus *Acartia* Dana, 1846**

**Subgenus Odontacartia Steurer, 1915**

##### Acartia nadiensis sp. nov.

Taxon classificationAnimaliaCalanoidaAcartiidae

6B2D92AF-5283-55D5-8C12-4ADE57FCB614

http://zoobank.org/DD2852BB-7AAE-4B65-85F1-4A741FD85F7F

Figures 1 , 2 , 3 , 4 , 5 , 6 , 7 , 8

###### Type locality.

Coastal water (17°45.848'S, 177°22.348'E), Nadi Bay, Fiji.

###### Materials examined.

All specimens have been deposited in the Marine Biodiversity Institute of Korea (MABIK). Holotype 1♀ (MABIK CR00246502) and Allotype 1♂ (MABIK CR00246503) undissected and preserved in 70% ethanol. Paratype: 2♀♀ (MABIK CR00246504-CR00246505) dissected on 13 and 10 slides, respectively; 2♂♂ (MABIK CR00246506-CR00246507) dissected on 14 and 8 slides, respectively; 10♀♀ (MABIK CR00246508-CR00246517) and 4♂♂ (MABIK CR00246518-CR00246521) undissected and preserved in 70% ethanol. 4♀♀ and 4♂♂ dried, mounted on stub, and coated with platinum for SEM. All specimens are from the type locality and were collected by S. Lee on 10 October 2013. The illustrations are based on the paratypes (♀, MABIK CR00246504; ♂, MABIK CR00246506).

###### Etymology.

The specific name refers to the type locality of Nadi bay, Fiji.

###### Description of female.

**Figure 1.**
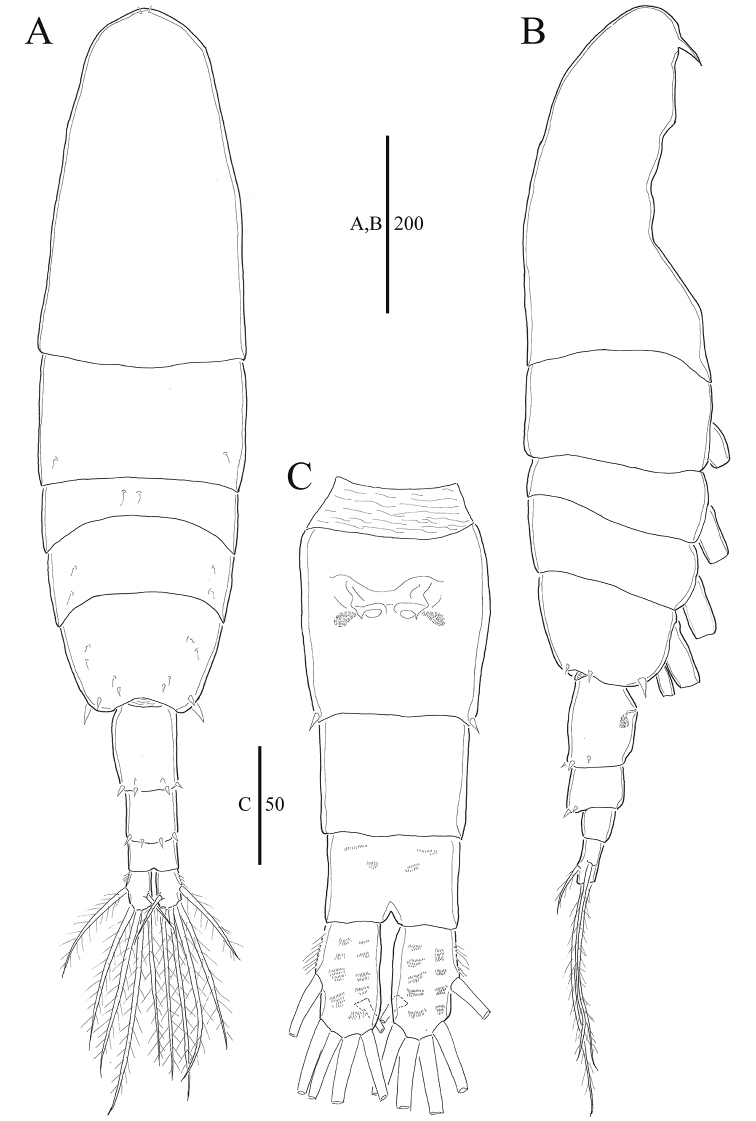
*Acartia nadiensis* sp. nov. female. **A** Habitus, dorsal **B** habitus, lateral **C** urosome, ventral. Scale bars: in μm.

Total body length 975–1050 μm (mean ± SD = 1018 ± 26 μm, *n* = 10, holotype 1015 μm) as measured from anterior margin of cephalosome to posterior margin of the caudal rami. Body surface armed with some sensillae (Fig. 1A). Prosome:urosome length ratio = 3.52:1.

**Figure 2.**
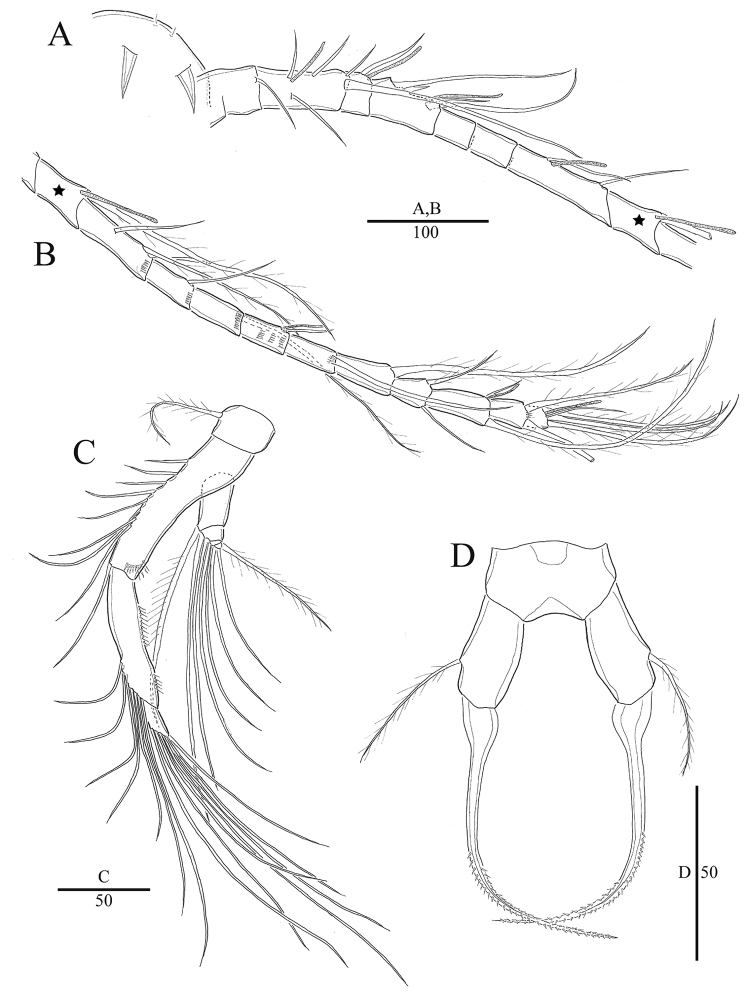
*Acartia nadiensis* sp. nov. female. **A** Rostrum and antennule (part, 1^st^ to 8^th^ segment) **B** antennule (part, 8^th^ to 18^th^ segment) **C** antenna **D** P5. Scale bars: in μm.

Prosome 5-segmented (Fig. 1A, B), cephalosome and first pedigerous somite completely separate; fourth and fifth pedigerous somite fused. Posterior corners of fifth pedigerous somite rounded, each with three spines. Rostral filaments thick and short (Figs 2A, 7A).

Urosome 3-segmented (Figs 1A–C, 7D–H, 8A), genital double somite slightly swollen anterolaterally, with paired gonopores ventromedially, each gonopore covered with pointed operculum; first and second urosomites each with four spines on posterodorsal margin. Caudal rami bearing short hairs on lateral margin. Proportional lengths of urosomites and caudal rami as 38:23:17:22 = 100.

Antennule incompletely 18-segmented (Fig. 2A, B), fourth to seventh segments partly fused on dorsal surface; ninth to eleventh segment each with one row of setules, twelfth segment with three rows of setules, thirteenth and seventeenth segment each with one row of setules; segmentation and setation patterns as follows: (1) I-[1], (2) II-VI-[5+ae], (3) VII-[1+ae], (4) VIII-XI-[4(1spiniform)+ae], (5) XII-[0], (6) XIII-[0], (7) XIV-XV-[2+ae], (8) XVI-[1+ae], (9) XVII-XVIII-[2+ae], (10) XIX-[1], (11) XX-[1], (12) XXI-[1+ae], (13) XXII-[1], (14) XXIII-[1], (15) XXIV-[2(1+1)], (16) XXV-[2(1+1)+ae], (17) XXVI-[2(1+1)], (18) XXVII-XXVIII-[4+ae].

Antenna (Fig. 2C): coxa with seta; basis and first endopodal segment fused to form elongated allobasis bearing eight setae medially and one seta terminally along inner marin, and spinular row on distal area; second endopodal segment elongated, with seven setae, rows of spinules on lateral margin; third exopododal segment short, with seven setae. Exopod 4-segmented; setation formula 1, 2, 2, 3.

**Figure 3.**
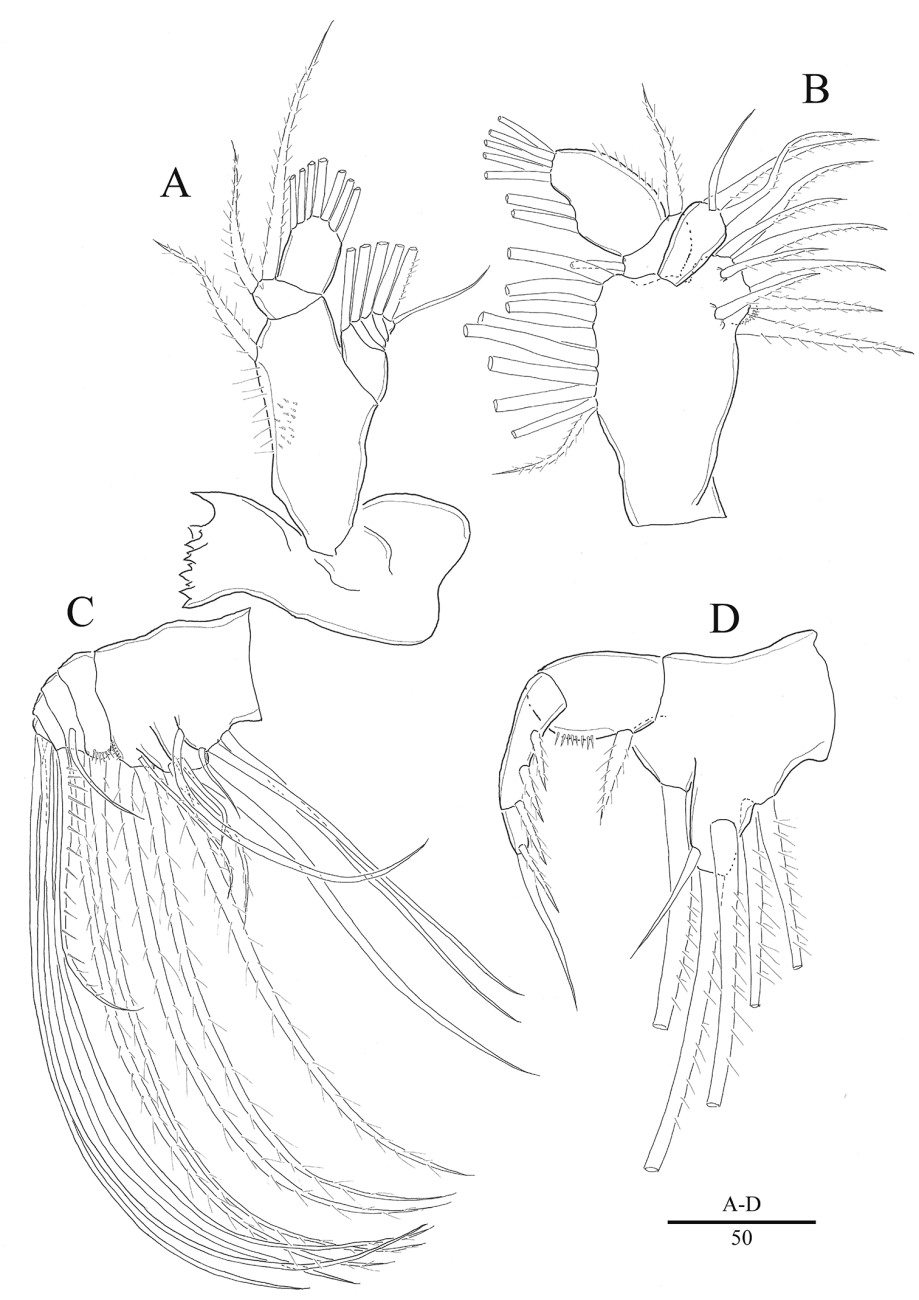
*Acartia nadiensis* sp. nov. female. **A** Mandible **B** maxillule **C** maxilla **D** maxilliped. Scale bars: in μm.

Mandible: (Fig. 3A) coxa with well developed gnathobase bearing eleven teeth; basis with seta and row of setules on lateral and posterior margins; endopod 2-segmented, first endopodal segment with two setae, second segment with seven setae; exopod 5-segmented, setation formula as 1, 1, 1, 1, 2.

Maxillule: (Fig. 3B) precoxa and coxa incompletely fused, praecoxal arthrite with eight setae; coxal endite with three setae; one short seta and eight long setae on coxal epipodite; basal endite with one seta; basal exite with one seta; 1-segment exopod with two setae laterally and five setae terminally; endopod absent.

Maxilla: (Fig. 3C) precoxa and coxa incompletely fused, setation formula of endites 4, 2, 2, 3; basal endite with a seta and row of spinules on distal margin; endopod 3-segmented, with setation formula 2, 2, 3.

Maxilliped (Fig. 3D) comprising syncoxa with six setae; basis with spiniform seta; endopod 2-segmented, first segment with three setae, second segment with two setae.

**Figure 4.**
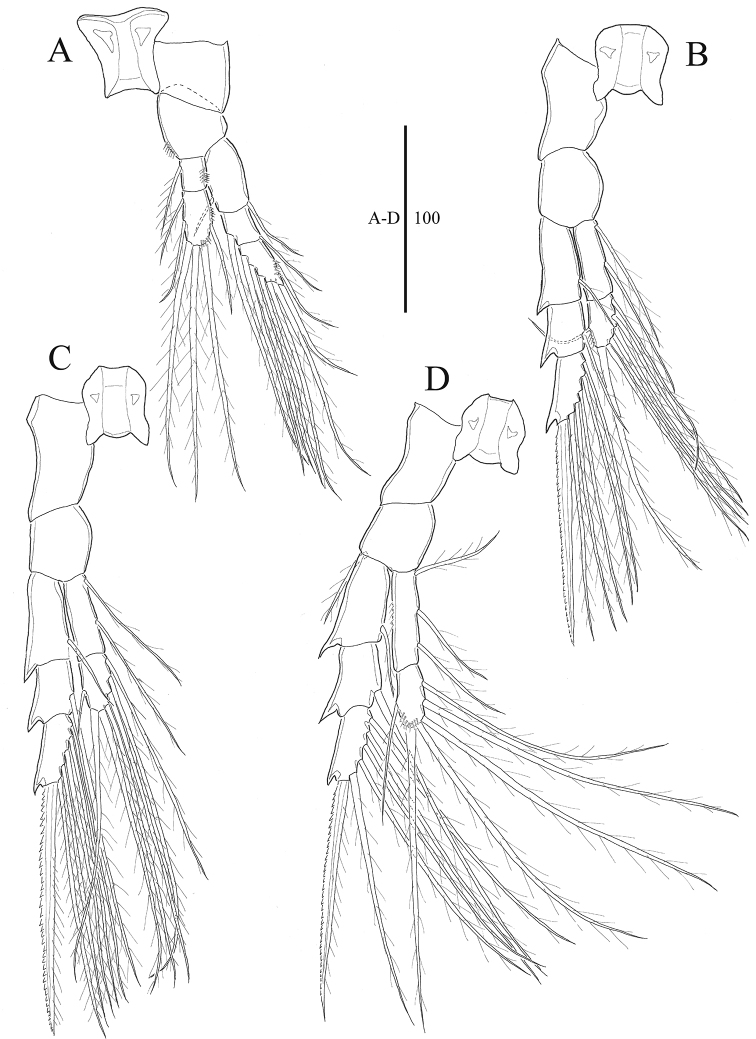
*Acartia nadiensis* sp. nov. female. **A** P1 **B** P2 **C** P3 **D** P4. Scale bars: in μm.

Legs 1–4 (Fig. 4A–D) biramous, each with 3-segmented exopod and 2-segmented endopod, and spinules along inner and outer margins as illustrated. Intercoxal sclerites well developed. Spine and setal formulae as follows:

P5 (Figs 2D, 7B, C) symmetrical, 3-segmented; basis ovate, with outer seta; exopod tapering, thick, bent at midlength, distal portion serrated, base slightly swollen.

###### Description of male.

**Figure 5.**
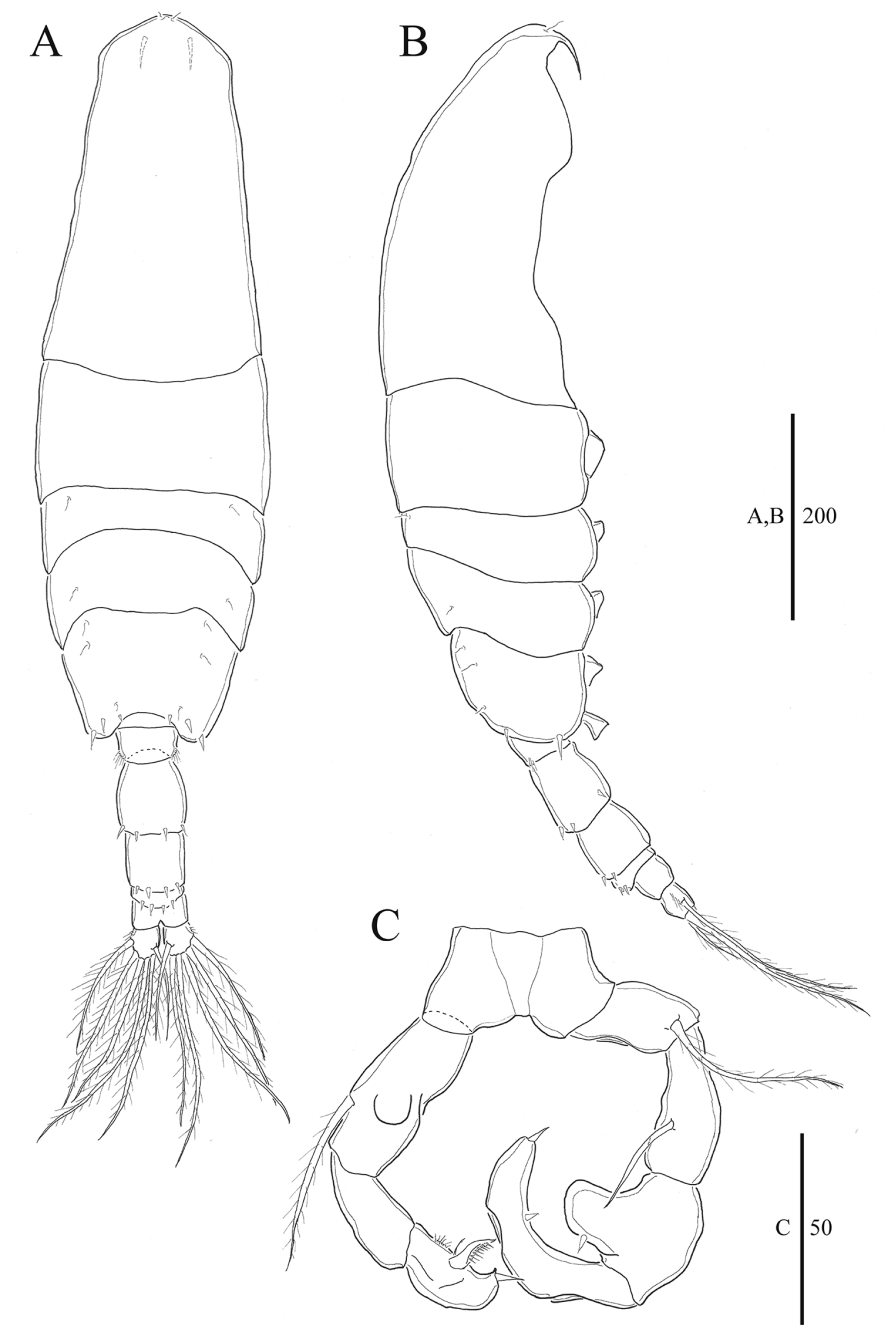
*Acartia nadiensis* sp. nov. male. **A** Habitus, dorsal **B** habitus, lateral **C** P5. Scale bars: in μm.

Total body length 910–952 μm (mean ± SD = 931 ± 16 μm, *n* = 5, allotype 930 μm) measured from anterior margin of cephalosome to posterior margin of caudal rami. Body surface armed with some sensilla (Fig. 5A, B). Prosome:urosome length ratio = 3.12:1.

Prosome (Fig. 5A, B) 5-segmented. Rostral filaments thin (Figs 5A, B, 8B). Fifth prosomite with six spines on posterior margin.

**Figure 6.**
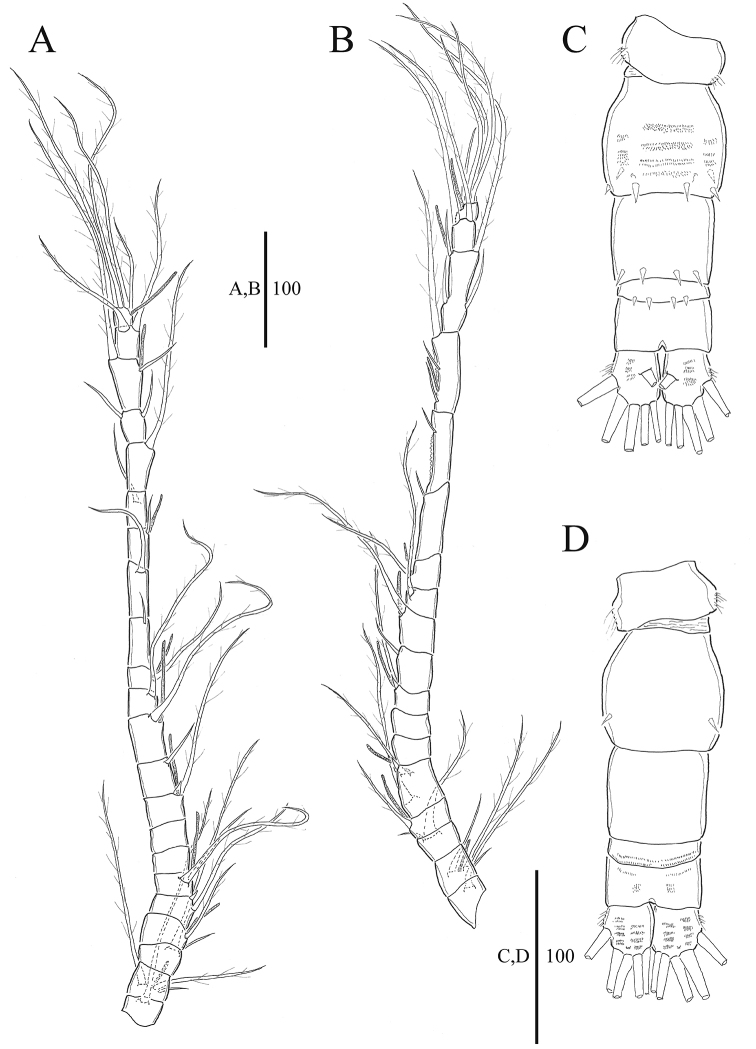
*Acartia nadiensis* sp. nov. male. **A** Antennule (left) **B** antennule (right) **C** urosome, dorsal **D** urosome, ventral. Scale bars: in μm.

Urosome (Figs 6C, D, 8C–H) 5-segmented. Second urosomite with four spines on posterodorsal margin and two spines on posteroventral margin; pair of sensillae on dorsal surface. Third and fourth urosomites each with four spines on posterodorsal margin. Caudal rami bearing short hairs on lateral margin. Length proportions of urosomites to caudal rami as 16:31:21:7:12:14 = 100.

Left antennule 22-segmented (Fig. 6A). Segmentation and setation pattern as follows: (1) I-[1], (2) II-VII-[3+ae], (3) VIII-[2], (4) IX-[1+ae], (5) X-[2(1spinifrom)], (6) XI-[2+ae], (7) XII-[0], (8) XIII-[0], (9) XIV-[2(1spiniform)+ae], (10) XV-[1], (11) XVI-[1+ae], (12) XVII-[1], (13) XVIII-[1+ae], (14) XIX-[1], (15) XX-[1], (16) XXI-[1+ae], (17) XXII-[1], (18) XXIII-[1], (19) XXIV-[2(1+1)], (20) XXV-[2(1+1)+ae], (21) XXVI-[2(1+1)], (22) XXVII-XXVIII-[4+ae]. Right antennule 18-segmented (Fig. 6B), with geniculation with fourteenth and fifteenth segments. Segmentation and setation pattern as follows: (1) I-[1], (2) II-VII-[3+ae], (3) VIII-[2], (4) IX -[1+ae], (5) X-XI-[3(1spiniform)+ae], (6) XII-[0], (7) XIII-[0], (8) XIV-[2(1spiniform)+ae], (9) XV-[1], (10) XVI-[1+ae], (11) XVII-[1], (12) XVIII-[1+ae], (13) XIX-[1], (14) XX-[1], (15) XXI-XXIII-[3+ae], (16) XXIV-XXV-[4(2+2)+ae], (17) XXVI-[2(1+1)], (18) XXVII-XXVIII-[4+ae].

Other mouthparts and P1–P4 as in female. P5 asymmetrical (Fig. 5C); intercoxal sclerite distinct. Left leg 4-segmented; basis armed with posterolateral seta and rounded lobe on posterior surface; exopod 2-segmented, exp-1 unarmed; exp-2 with hairs, and one spine with teeth on medial margin and one small spine distally. Right leg 5-segmented, basis armed with posterolateral seta. Exopod 3-segmented, exp-1 with long slender seta; exp-2 with oblong inner lobe bearing one spine on distal margin; exp-3 with one spine on medial margin and one spine distally.

**Figure 7.**
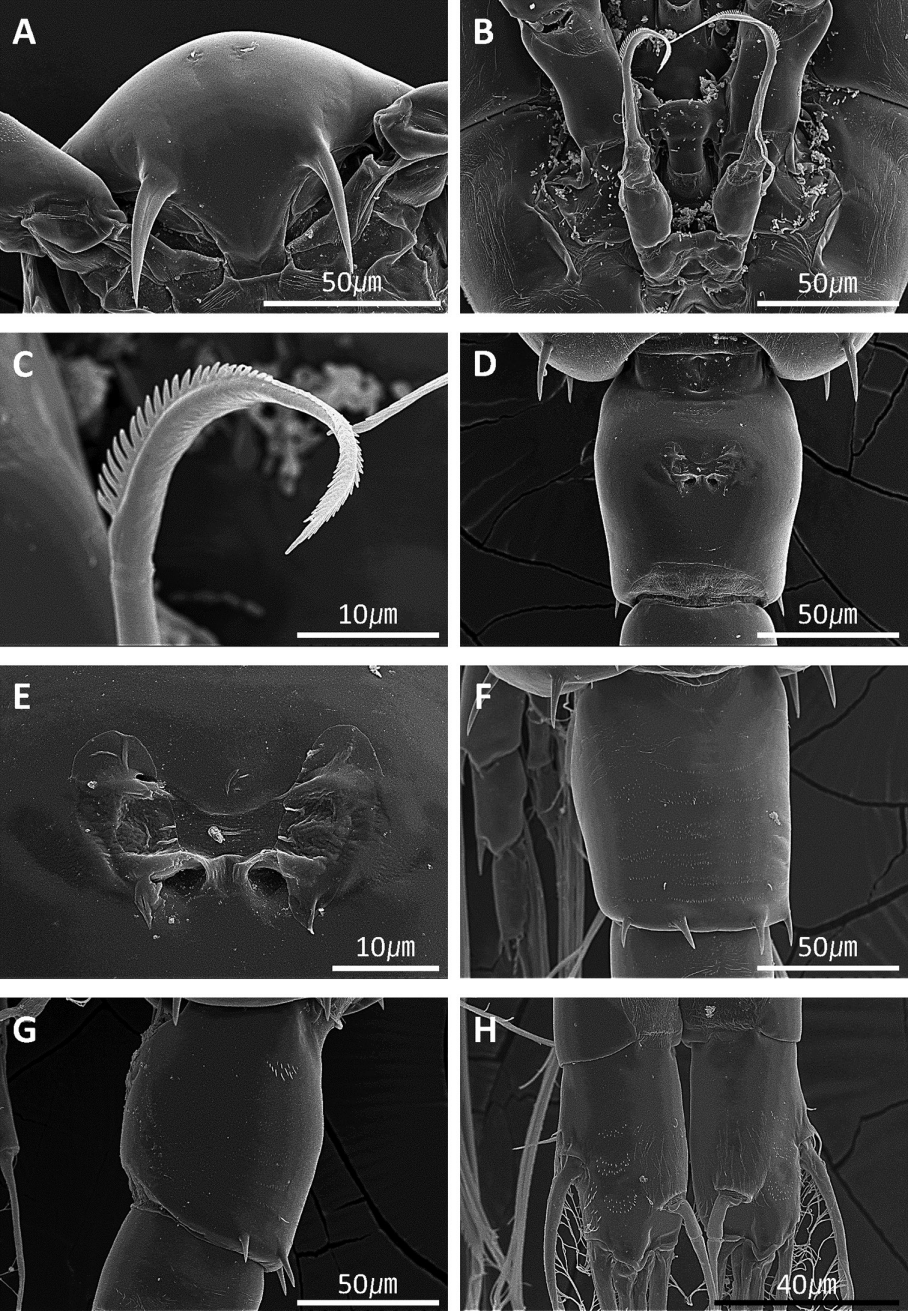
*Acartia nadiensis* sp. nov. Scanning electron micrographs. **A** Female, rostrum **B** female, P5 **C** female, P5, terminal spine **D** female, genital double-somite **E** female, genital field **F** female, 1^st^ urosomite, dorsal view **G** female, 2^nd^ urosomite, lateral view **H** female, caudal rami, dorsal view. Scale bars: in μm.

**Figure 8.**
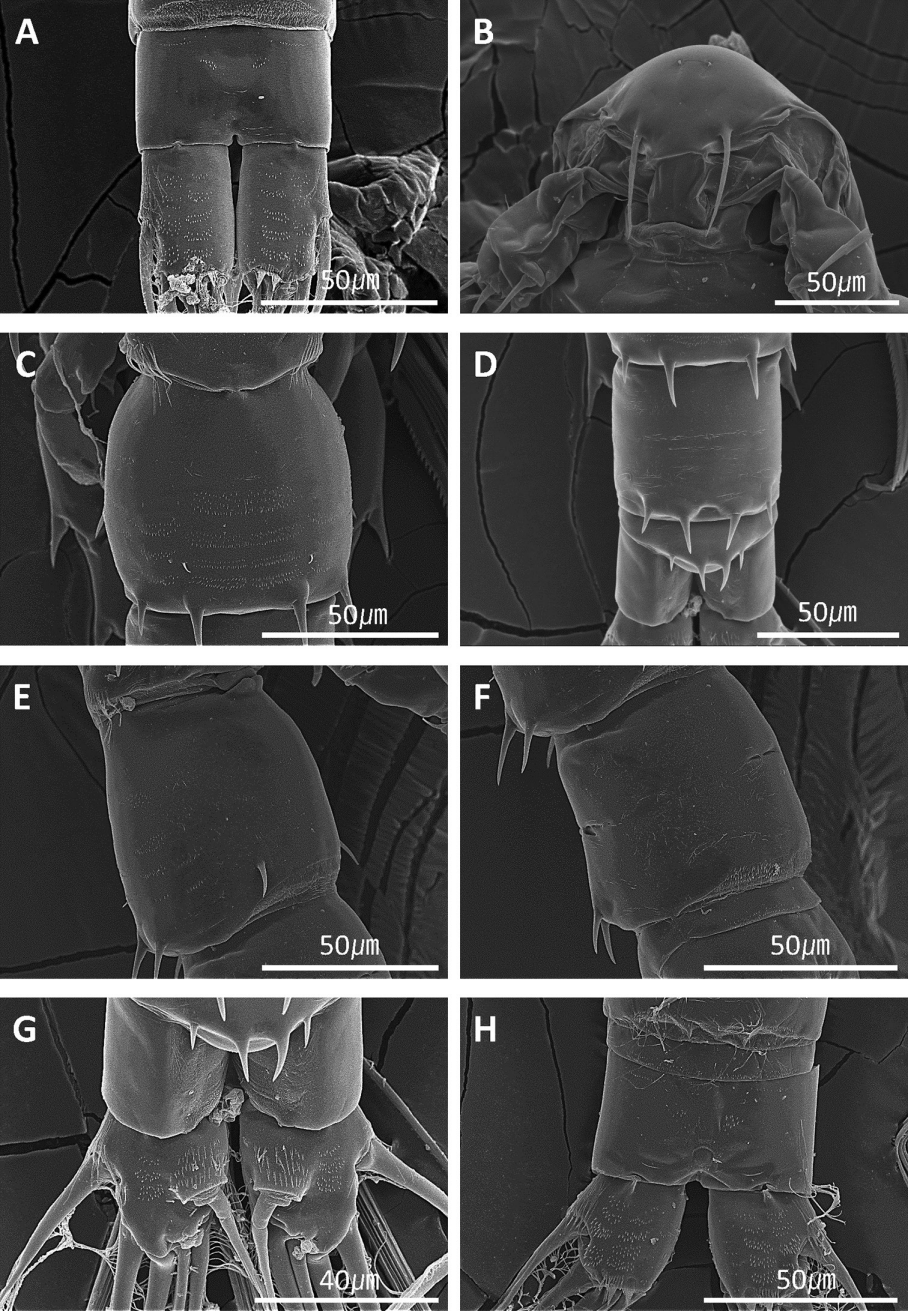
*Acartia nadiensis* sp. nov. Scanning electron micrographs. **A** Female, urosome and caudal rami, ventral view **B** male, rostrum **C** male, 1^st^ urosomite **D** male, 2^nd^–4^th^ urosomite, dorsal view **E** male, 1^st^ urosomite, lateral view **F** male, 2^nd^ urosomite, lateral view **G** male, 5^th^ urosomite and caudal rami, dorsal view **H** male, 4^th^ urosomite and caudal rami, ventral view. Scale bars: in μm.

## Molecular analysis

**Figure 9.**
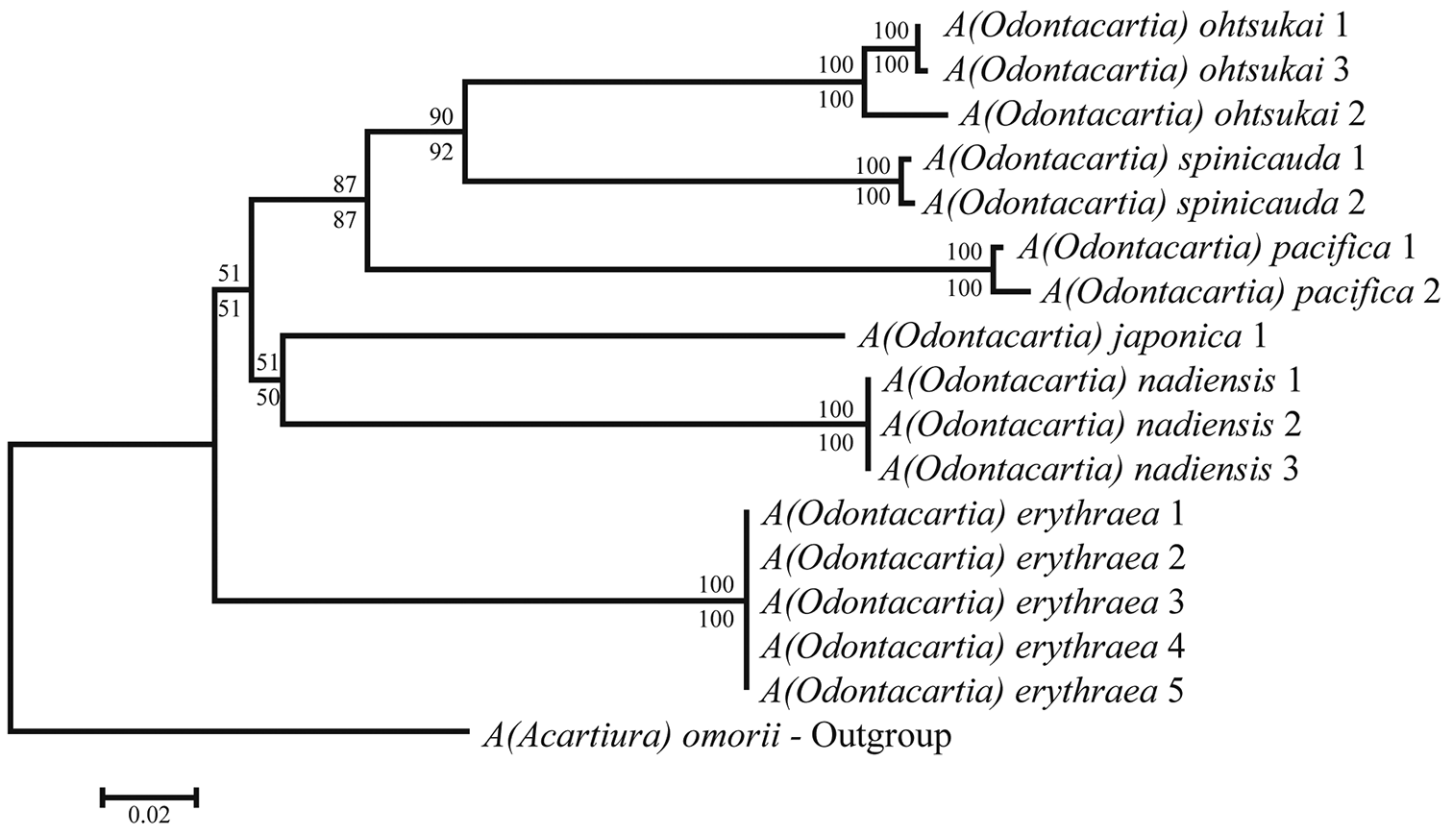
Phylogenetic tree based on mtCOI sequences (581 bp) of *Odontacartia* species including A. (Acartiura) omorii as outgroup. One-thousand bootstrap replicates were performed by MEGA6 using neighbor joining and minimum evolution methods. Neighbor joining bootstrap values shown above branches; minimum evolution bootstrap values are below branches.

A 581 bp partial region of mtCOI was sequenced from five species: *A. nadiensis* sp. nov., *A. erythraea*, *A. japonica*, *A. ohtsukai*, and *A. omorii*. Sequences of two species (*A. pacifica* and *A. spinicauda*) were obtained from NCBI and also included in the analysis. All species belong to the subgenus Odontacartia except *A. omorii*, which belongs to the subgenus Acartiura and was used as the outgroup. The mtCOI sequences of *A. nadiensis* differed in a 24.1% from *A. japonica*, and in up to 29.0% from *A. pacifica* (Table 2). Neighbor joining and minimum evolution phylogenetic analyses using the Tamura-Nei model showed that *A. nadiensis* was clearly distinct from its congeneric species (Fig. 9).

**Table 2.** Genetic variation within the subgenus Odontacartia species based on mtCOI sequence comparison including A. (Acartiura) omorii as outgroup.

	1	2	3	4	5	6	7	8	9	10	11	12	13	14	15	16
**1.**A. (Odontacartia) nadiensis 1																
**2.**A. (Odontacartia) nadiensis 2	0.000															
**3.**A. (Odontacartia) nadiensis 3	0.000	0.000														
**4.**A. (Odontacartia) erythraea 1	0.247	0.247	0.247													
**5.**A. (Odontacartia) erythraea 2	0.247	0.247	0.247	0.000												
**6.**A. (Odontacartia) erythraea 3	0.247	0.247	0.247	0.000	0.000											
**7.**A. (Odontacartia) erythraea 4	0.247	0.247	0.247	0.000	0.000	0.000										
**8.**A. (Odontacartia) erythraea 5	0.247	0.247	0.247	0.000	0.000	0.000	0.000									
**9.**A. (Odontacartia) japonica 1	0.241	0.241	0.241	0.244	0.244	0.244	0.244	0.244								
**10.**A. (Odontacartia) ohtsukai 1	0.277	0.277	0.277	0.258	0.258	0.258	0.258	0.258	0.264							
**11.**A. (Odontacartia) ohtsukai 2	0.275	0.275	0.275	0.266	0.266	0.266	0.266	0.266	0.269	0.028						
**12.**A. (Odontacartia) ohtsukai 3	0.278	0.278	0.278	0.260	0.260	0.260	0.260	0.260	0.267	0.002	0.030					
**13.**A. (Odontacartia) pacifica 1	0.288	0.288	0.288	0.282	0.282	0.282	0.282	0.282	0.267	0.249	0.249	0.252				
**14.**A. (Odontacartia) pacifica 2	0.290	0.290	0.290	0.287	0.287	0.287	0.287	0.287	0.278	0.254	0.254	0.257	0.009			
**15.**A. (Odontacartia) spinicauda 1	0.266	0.266	0.266	0.254	0.254	0.254	0.254	0.254	0.292	0.183	0.203	0.181	0.252	0.262		
**16.**A. (Odontacartia) spinicauda 2	0.263	0.263	0.263	0.259	0.259	0.259	0.259	0.259	0.287	0.183	0.203	0.181	0.247	0.257	0.003	
**17.** A. (Acartiura) omorii	0.292	0.292	0.292	0.251	0.251	0.251	0.251	0.251	0.270	0.297	0.290	0.299	0.296	0.304	0.268	0.270

## Discussion

The new species, *Acartia nadiensis* sp. nov., clearly belongs to the subgenus Odontacartia. This subgenus displays the following diagnostic characters compared to the other five subgenera of *Acartia*: presence of rostral filaments, symmetrical pointed process on the posterior corner of the last prosomite, minutely serrated terminal spine on female P5, and absence of protrusion on the basis of male right P5 (Steuer 1915; Ueda and Bucklin 2006; Soh 2010). The new species can be distinguished from other *Odontacartia* species by several distinctive characters (Table 3). First, *Odontacartia* species, including the new species, can be divided into two groups based on the presence of a spine on the first segment of antennules (Steuer 1923; Srinui et al. 2019). Species with this spine include *A. amboinensis*, *A. australis*, *A. bispinosa*, *A. erythraea*, *A. japonica*, and *A. lilljeborgi*. Species lacking spine include *A. bowmani*, *A. centura*, *A. mertoni*, *A. ohtsukai*, *A. pacifica*, and *A. spinicauda*. *Acartia nadiensis* sp. nov. also lacks a spine on the first segment of antennules. Second, the outer seta of the female P5 of *A. nadiensis* sp. nov. is much shorter than the terminal spine, and the length ratio of the outer seta/terminal spine is 0.4. Most species of *Odontacartia* have an outer seta that is longer than the terminal spine in female P5. There are two species (*A. bowmani* and *A. japonica*) that have a short terminal seta on female P5, and the length ratio of outer seta/terminal seta are 0.9 and 0.7, respectively. Third, the male P5 of *A. nadiensis* is clearly distinguishable from the rest of species based on its length and the type of medial process on the exp-2 of the left leg. Furthermore, the new species shows other minor differences compared to the other 13 *Odontacartia* species, such as the number of dorsal spines on the urosomite, the length/width ratio of the female P5 basis, and the length/width ratio of caudal rami.

**Table 3.** Morphological differences among species within the subgenus Odontacartia (Calanodia: Acartiidae: *Acartia*).

	***A. nadiensis* sp. nov.**	***A. amboinensis***	***A. australis***	***A. bispinosa***	***A. bowmani***	***A. centura***	***A. edentata***	***A. erythraea***	***A. japonica***	***A. lilljeborgi***	***A. mertoni***	***A. ohtsukai***	***A. pacifica***	***A. spinicauda***
**Female**														
Body length	975–1050	1340–1510	1290–1400	1320–1530	1200–1300	1350–1400	1190–1230	1400	1350–1410	1330–1400	ND	1190–1230	1190–1210	1250
Spine on 1^st^ seg of antennules	absent	present	present	present	absent	absent	absent	present	present	present	absent	absent	absent	absent
P5														
Basis length/width ratio	2	4	2.5	2.4	1.5	1.6	1.4	2.3	2.1	1.4	2	1.8	1.4	1.4
Length ratio of P5 outer seta/terminal spine	0.4	1.8	1.2	1.4	0.9	1.6	1.3	1.6	0.7	1.5	1	1	1.8	1.2
Urosome														
Dorsal spines on 1^st^ urosomite	4	2	2	2	0	2	0	2	2	(small spinules)	2	2	2	2
Dorsal spines on 2^nd^ urosomite	4	4	0	0	2	2	2	2	(small spinules)	(small spinules)	2	2	2	2
Caudal rami length/width ratio	1.8	1.3	1.1	1.8	2	1.7	3	1.4	1.2	1.5	2	3	2.5	3
**Male**														
Body length	910–952	ND	1170–1230	1070–1160	1100	1250–1280	1080–1150	ND	1190–1240	1100	ND	1030–1050	1030–1160	ND
Left P5														
Length ratio of medial process/segment on 2^nd^ exopodite	0.5	ND	0.7	0.4	0.9	0.8	2	0.4	1	0.5	3.5	1.4	1.6	0.9
Type of medial process on 2^nd^ exopodite	Spine with teeth	ND	Spine	Spine with fine setae	Spine	Spine	Long seta	Spine	Spine with teeth	Spine	Long seta	Long seta	Long seta	Spine
**References**	This study	Tanaka 1965	Ueda 1986	Nishida 1985; El-Sherbiny and Al-Aidaroos 2014	Abraham 1976	Abraham 1976	Srinui et al. 2019	Mori 1964	Ueda 1986	Giesbrecht 1892	Steuer 1923; Ueda and Bucklin 2006	Ueda and Bucklin 2006	Ueda and Bucklin 2006	Giesbrecht 1892; Mori 1964

To supplement the morphological evidences, we conducted molecular phylogenetic analyses using partial mtCOI sequences of six *Odontacartia* species, including the new species. The mtCOI gene is widely used to identify sibling species due to its higher evolutionary rate than 16s and 18s rDNA (Knowlton and Weight 1998; Hebert et al. 2003; Schindel and Miller 2005; Karanovic et al. 2018). In previous studies of calanoid copepods, mtCOI sequence divergence between species have been shown to range from 13.0–22.0% (Bucklin et al. 1999), 17.6–26.7% (Eyun et al. 2007), and 21.0–23.0% (Soh et al. 2013). The mtCOI partial sequence of *A. nadiensis* sp. nov. differed by 24.1–29.0% from the sequences of congeneric species, which is greater than the range of interspecific differences reported in previous studies.

The length ratio of the outer seta/terminal spine of the female P5 is the most diagnostic morphological feature in *Odontacartia* species. However, this character is also used to determine the subgenus Euacartia (Soh et al. 2013). This confusion between subgenus systems has been documented previously (Madhupratap and Haridas 1994). Barthélémy (1999) compared female genital structure of 25 species of Acartiidae using light and scanning electron microscopy and concluded that there is no support for the current subdivision of *Acartia* into subgenera. Although the new species *A. nadiensis* belongs to the subgenus Odontacartia based on the current identification system, the validity subgeneric taxa, as proposed by Steuer (1915, 1923), within *Acartia* should be reevaluated.

## Key to species of the subgenus Odontacartia Steuer, 1915

**Table:** 

1	Presence of spine on 1^st^ to 2^nd^ segments of female antennule	**2**
–	Absence of spine on 1^st^ to 2^nd^ segments of female antennule	**5**
2	Small spinule row present on dorsal surface of female 1^st^ urosomite	***A. lilljeborgi***
–	Strong spines present on dorsal surface of female 1^st^ urosomite	**3**
3	Absence of processes (spines and spinules) on dorsal surface of female 2^nd^ urosomite	**4**
–	Small spinule row present on dorsal surface of female 2^nd^ urosomite	***A. japonica***
–	2 strong spines present on dorsal surface of female 2^nd^ urosomite	***A. erythraea***
–	4 strong spines present on dorsal surface of female 2^nd^ urosomite	***A. amboinensis***
4	Length-width of female caudal rami are almost similar; medial process on 2^nd^ exopodite of male left P5 as spine	***A. australis***
–	Female caudal rami almost twice longer than wide; medial process on 2^nd^ exopodite of male left P5 as spine with fine seta	***A. bispinosa***
5	Dorsal surface of female 1^st^ urosomite devoid of processes (spines and spinules)	**6**
–	Spine present on dorsal surface of female 1^st^ urosomite	**7**
6	Female caudal rami twice longer than wide; medial process and 2^nd^ exopodite segment of male left P5 almost similar in length	***A. bowmani***
–	Female caudal rami three times longer than wide; medial process of male left P5 twice longer than 2^nd^ exopodite segment	***A. edentata***
7	Dorsal surface of female 1^st^ and 2^nd^ urosomite with two strong spines	**8**
–	Four strong spines on dorsal surface of female 1^st^ and 2^nd^ urosomite	***A. nadiensis* sp. nov.**
8	Length of female P5 outer seta and terminal spine similar	**9**
–	Female P5 outer seta is longer than terminal spine	**10**
9	Female caudal rami is twice as long as wide	***A. mertoni***
–	Female caudal rami three times longer than wide	***A. ohtsukai***
10	Length-width ratio of female caudal rami as 1.7; medial process on 2^nd^ exopodite male left P5 as spine	***A. centura***
–	Length-width ratio of female caudal rami as 2.5; medial process on 2^nd^ exopodite of male left P5 as long seta	***A. pacifica***
–	Length-width ratio of female caudal rami as 3; medial process on the 2^nd^ exopodite of male left P5 as spine	***A. spinicauda***

## Supplementary Material

XML Treatment for Acartia nadiensis
